# Mitochondrial depletion syndrome type 3: the Lebanese variant

**DOI:** 10.3389/fgene.2023.1215083

**Published:** 2023-06-29

**Authors:** Marianne Majdalani, Nadine Yazbeck, Lamis El Harake, Jinane Samaha, Pascale E. Karam

**Affiliations:** ^1^Division of Pediatric Intensive Care Unit, Department of Pediatrics and Adolescent Medicine, American University of Beirut Medical Center, Beirut, Lebanon; ^2^Division of Gastroenterology, Department of Pediatrics and Adolescent Medicine, American University of Beirut Medical Center, Beirut, Lebanon; ^3^ Faculty of Medicine, American University of Beirut, Beirut, Lebanon; ^4^ Inherited Metabolic Diseases Program, Department of Pediatrics and Adolescent Medicine, American University of Beirut Medical Center, Beirut, Lebanon

**Keywords:** *DGUOK*, mitochondrial depletion, mtDNA maintenance defects, MENA, neonatal liver failure, lactic acidosis, liver transplantation, Lebanon

## Abstract

**Introduction:** Mitochondrial DNA depletion syndrome type 3 is an emerging disorder linked to variants in the deoxyguanosine kinase gene, which encodes for mitochondrial maintenance. This autosomal recessive disorder is frequent in the Middle East and North Africa. Diagnosis is often delayed due to the non-specificity of clinical presentation with cerebro-hepatic deterioration. The only therapeutic option is liver transplantation, although the value of this remains debatable.

**Methods:** We describe the clinical, biochemical, and molecular profiles of Lebanese patients with this rare disorder. We also present a review of all cases from the Middle East and North Africa.

**Results:** All Lebanese patients share a unique mutation, unreported in other populations. Almost half of patients worldwide originate from the Middle East and North Africa, with cases reported from only 7 of the 21 countries in this region. Clinical presentation is heterogeneous, with early-onset neurological and hepatic signs. Liver failure and lactic acidosis are constants. Several variants can be identified in each population; a unique c.235C>T p. (Gln79*) pathogenic variant is found in Lebanese patients. Outcome is poor, with death before 1 year of age.

**Conclusion:** The pathogenic nonsense variant c.235C>T p. (Gln79*) in the deoxyguanosine kinase gene may be considered a founder mutation in Lebanon. Further genotypic delineation of this devastating disorder in populations with high consanguinity rates is needed.

## 1 Introduction

Mitochondrial DNA (mtDNA) maintenance defects are emerging genetic disorders encompassing multiple mtDNA deletion and mtDNA depletion syndromes (MTDSs) resulting from qualitative and quantitative defects in mtDNA synthesis, respectively. Depending on the underlying enzymes involved, mtDNA maintenance defects are categorized into several types of disorder: mtDNA synthesis, mitochondrial nucleotide salvage pathway, cytosolic nucleotide metabolism, mitochondrial nucleotide import, and mitochondrial fusion disorders. These rare disorders are caused by pathogenic variants in one of the 20 nuclear genes encoding proteins implicated in mtDNA maintenance (El-Hattab et al., 2018). They are characterized by broad phenotypic variability, reflecting the genotypic variants and the combination of organs involved. Mitochondrial DNA depletion syndromes, first described by Mandel in 2001, can be grouped into myopathic, cardiomyopathic, encephalomyopathic, neurogastrointestinal, and hepatocerebral types (Basel, 2020). The deoxyguanosine kinase (*DGUOK*) gene is a nuclear gene, mapped to chromosome 2p13 (Johansson et al., 1996), encoding for mitochondrial deoxyribonucleoside triphosphate synthesis. *DGUOK* deficiency decreases the mtDNA copy number and impairs the activity (Freisinger et al., 2006) and synthesis (Mandel et al., 2001) of the respiratory chain complexes (I, III, IV, and V). Clinically, the age of onset may vary from the neonatal and early infancy period to childhood. The early-onset form, or MTDS type 3 (OMIM 251880), is autosomal–recessively inherited and is linked to bi-allelic pathogenic variants of the *DGUOK* gene. It represents the only hepato-cerebral type of mtDNA maintenance defect related to the mitochondrial nucleotide salvage pathway (Sezer et al., 2015; El-Hattab et al., 2018).

Most MTDS type 3 patients show multisystem involvement, with a variable combination of digestive and neurologic manifestations associated with impaired growth (Basel, 2020). Antenatal energy deficit is reflected in microcephaly, low birth weight, and intra-uterine growth retardation. Neonates or infants may present with any constellation of the following symptoms: vomiting, jaundice, hepato/splenomegaly, ascites, and progressive liver failure, as well as hypotonia, nystagmus, seizures, and developmental delay (El-Hattab and Scaglia, 2009).

Diagnosis is often delayed due to the variable and non-specific clinical presentation of MTDS type 3, which may mimic other inherited metabolic diseases such as hereditary tyrosinemia type I and classical galactosemia (Demirbas et al., 2018), in addition to non-metabolic infectious diseases, mainly viral infections such as cytomegalovirus and herpes simplex virus (Karadağ et al., 2021). Biochemical testing may reveal lactic acidosis and hypoglycemia from the first week of life, and later increased liver enzymes, followed by progressive hepatic cytolysis and coagulopathy. MTDS type 3 can be suspected upon detection (via tandem mass spectrometry screening of newborns) of elevated plasma tyrosine and phenylalanine, with normal succinylacetone levels. Plasma amino acids chromatography confirms hypertyrosinemia and hyperphenylalaninemia as well as hyperalaninemia, reflecting hepatic insult and lactic acidosis, respectively (Mudd et al., 2012). The final diagnosis of MTDS type 3 is confirmed by genetic testing. Theoretically, a multigene panel or even single-gene sequencing would be sufficient to identify *DGUOK* gene mutations; however, whole-exome sequencing is more frequently used by physicians to reach the diagnosis in view of the non-specificity of the presenting clinical symptoms. To date, per the Human Gene Mutation Database, 57 disease-causing variants in the *DGUOK* gene are known, with most being missense and nonsense mutations. Outcome is usually poor, with death occurring before 4 years of age (Dimmock et al., 2008). Treatment is symptomatic, with heterogeneous responses to liver transplantation (Jankowska et al., 2021).

More than 100 patients with MTDS type 3 have been reported so far (El-Hattab et al., 2017). Cases from the Middle East and North Africa (the MENA region) account for almost half of these, which reflects the high consanguinity rates producing a predisposition to autosomal recessive disorders. Reports are available of patients from North Africa (Labarthe et al., 2005; Brahimi et al., 2009; AlMenabawy et al., 2022), from Saudi Arabia (Al-Hussain et al., 2014), from Iran (Mahjoub et al., 2019), and in one cohort of Israeli Druze (Mandel et al., 2001). Only two Lebanese patients, carrying the nonsense *DGUOK* mutation c.235C>T p. (Gln79*), have previously been reported [by Mancuso et al. (2005)].

We describe the clinical, biochemical, and molecular profiles of three other Lebanese patients with this unique *DGUOK* variant, which remains unreported in other populations.

## 2 Patients and methods

A retrospective review of the charts of patients diagnosed with MTDS type 3 at the American University of Beirut Medical Center (AUBMC) was conducted. The collected data included initial clinical manifestations; biochemical, radiological, and genetic investigations; and outcome. For genetic testing, samples were sent to Centogene laboratory, Germany, for next-generation sequencing. The generated library was sequenced, at this reference laboratory, on an Illumina platform to obtain at least 20× coverage depth for >98% of the targeted bases. At Centogene, variants with low quality and/or unclear zygosity are confirmed by orthogonal methods. Consequently, specificity of >99.9% for all reported variants is achieved.

An analysis of clinical, biochemical, and molecular data for all MTDS type 3 patients from the MENA region, including the Lebanese patients, is also presented.

Written informed consent was obtained from the legal guardians/parents of the patients for participation in this study and for the publication of any potentially identifiable data included in this article, in accordance with our institutional requirements and the Declaration of Helsinki.

## 3 Results

### 3.1 Patients

**FIGURE 1:**
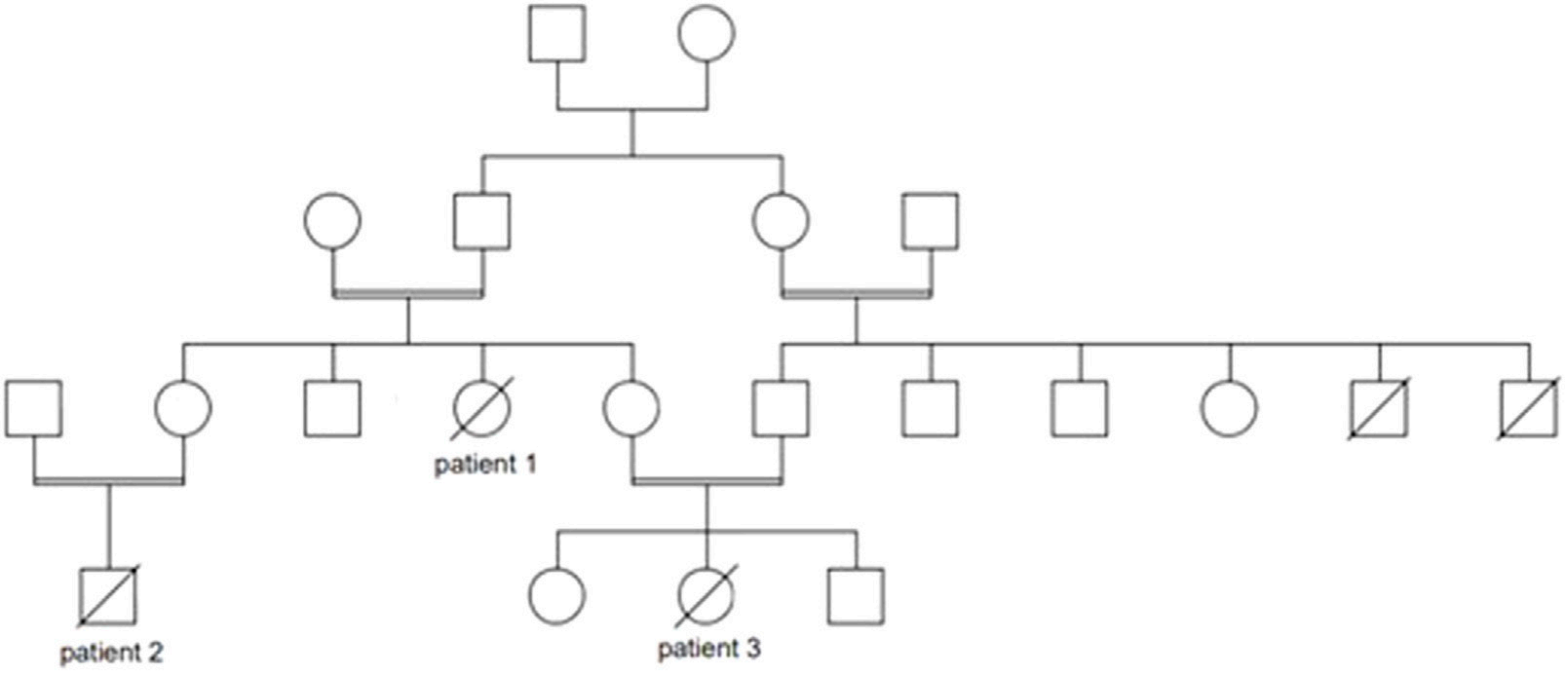
Pedigree of the Lebanese patients with mitochondrial depletion syndrome type 3.

**Patient 1** was a female infant born at term, the product of 39 weeks of gestation, to a primigravida 29-year-old mother, with a birth weight of 1855 g, length 44 cm, and head circumference 31 cm; all parameters were below 1% No perinatal complications were noted. Parents were consanguineous, with positive family history of death in early infancy of two male cousins with undiagnosed liver disease (Figure 1). The patient was admitted to the neonatal intensive care for symmetrical intrauterine growth retardation. On physical examination, she exhibited microcephaly, nystagmus, hepatomegaly, and severe hypotonia. In the immediate neonatal period, she developed hypoglycemia (32 mg/dl). Laboratory tests showed elevated liver enzymes and cholestasis (Table 1). The results of sepsis work-up were negative. Viral serologies were also negative. Metabolic work-up (Table 1) confirmed the presence of lactic acidosis and hypertyrosinemia. Neonatal screening via tandem mass spectrometry was positive for elevated plasma tyrosine, with normal succinylacetone and galactose-1-phosphate uridyltransferase activity. Urine organic acids chromatography was suggestive of liver failure with lactic acidosis (Table 1). Brain magnetic resonance spectroscopy revealed a small lactate peak in the left frontal and right parietal lobes at 1.3 ppm. A mitochondrial hepatopathy was suspected. Genetic testing could not be carried out for financial reasons. The patient required several infusions of fresh frozen plasma and cryoprecipitate for acute liver failure. She was discharged at 1 month of age with the same cerebro-hepatic symptoms noted at birth. Muscle and liver biopsies were offered to advance the diagnosis, but the family declined. On follow-up visits every 3 months, she was observed to have microcephaly with severe failure to thrive (growth parameters below 3%) and psychomotor delay. At 7 months of age, her neurological examination was stable, with head lag, axial hypotonia, and nystagmus. She had persistent lactic acidosis and liver cytolysis, cholestasis, and coagulopathy. She passed away at another hospital, undiagnosed, at the age of 11 months from liver failure and gastrointestinal bleed.

**TABLE 1:** Initial biochemical investigations of three Lebanese patients with mitochondrial depletion syndrome type 3. Abbreviations: AST, aspartate aminotransferase; ALT, alanine aminotransferase; GGT, gamma-glutamyl transferase; AFP, alpha-fetoprotein; INR, international normalized ratio; TMS, tandem mass spectrometry; GALT, galactose-1-phosphate uridyltransferase. *age; m, months; w, weeks.

		Test (*reference range*)	Patient 1 (2w)*	Patient 2 (2 m)*	Patient 3 (3w)*
Initial investigations	*Liver function tests*	Glucose (*76–110 mg/dl*)	32	19	33
		AST (*0–50 IU/L*)*/*ALT (*0–85 IU/L*)	102/72	180/98	84/60
		GGT (*10–50 IU*)	579	68	461
		Bilirubin total/direct (*0–1.2/0–0.3 mg/dl*)	12.8/1.2	10.8/7.7	1.3/1.2
		AFP (*5–250* *U/mL*)	61,754	86,805	92,476
		INR (0.9–1.2)	2.4	3.8	3.6
Metabolic work-up	*Basic tests*	Ammonia (*≤ 50 μmol/L*)	24	44	54
		Lactate (*0.55–2.20 mmol/L*)	4.46	5.9	7.32
	*Plasma amino acids chromatography*	Tyrosine (*55–147 μmol/l*) *at < 1* *m*	948	1810	394
		*(22–108 μmol/l) at 1* *m–2y*			
		Phenylalanine (*38–137 μmol/l*) *at < 1* *m*	96	229	65
		(31–75 *μmol/l*) *at 1* *m–2y*			
	*Newborn screening (TMS)*	Tyrosine (*20–450 μmol/l*)	1104	1632	621
		Succinylacetone (*<1.16 ng/ml*)	0.25	0.37	0.41
		GALT assay (*3.5–100 U/g Hb*)	9.5	7.5	5.1
	*Urine organic acids chromatography*	4-hydroxyphenyllactic acid (*<2 mmol/mol creatinine*)	1399	Increased	1047
		4-hydroxyphenylpyruvic acid (*<2 mmol/mol creatinine*)	236	Increased	166
		N-acetyltyrosine (*<2* *mmol/mol creatinine*)	54	Increased	42

**Patient 2**, maternal nephew of patient 1 (Figure 1), was a 2-month-old male infant, the product of a full-term pregnancy to first-degree consanguineous parents, with no perinatal complications. Proportionate intrauterine growth retardation was reported. He was born in a hospital where neonatal screening was not offered. The patient exhibited poor weight gain (300 g) during the first month of life, for which several formula milks were tried, including an amino acid formula for suspected cow’s milk protein allergy. At presentation to AUBMC, he exhibited failure to thrive (all parameters below 3%), generalized jaundice, hepatomegaly, nystagmus, and hypotonia. Laboratory investigations were suggestive of hepatopathy associated with lactic acidosis (Table 1). Whole-exome sequencing revealed a homozygous, pathogenic nonsense mutation in the *DGUOK* gene, c.235C>T p. (Gln79*), consistent with the genetic diagnosis of autosomal recessive MTDS type 3. Both parents were heterozygous carriers of this mutation. Sanger sequencing was not performed by Centogene, since the identified variant was of good quality. The patient passed away 1 month later, in septic shock and with severe lactic acidosis.

**Patient 3**, maternal niece of patient 1 and cousin of patient 2 (Figure 1), was a female infant born prematurely, to consanguineous parents, at 35 weeks 5 days of gestation due to maternal placenta previa. She exhibited symmetrical intrauterine growth retardation; her birth weight was 1900g, with all growth parameters below 3% Apgar scores were 8 at 1 min and 9 at 5 min. At 3 weeks of age, she was referred to the Inherited Metabolic Diseases Program at AUBMC for newborn screening, which was positive, indicating an elevated tyrosine level. She was mildly jaundiced, with hepatomegaly, microcephaly, nystagmus, hypotonia, and poor weight gain (200 g). She was found to have hypoglycemia and lactic acidosis with liver dysfunction, which warranted further metabolic investigations (Table 1). The familial MTDS type 3 was confirmed by whole-exome sequencing. Her parents were confirmed to be heterozygous carriers of the same mutation. In view of the good quality of the variant identified at the Centogene laboratory, Sanger sequencing was not performed. She subsequently presented at 2 months of age with recurrent vomiting and decreased feeding. She passed away, within 24 h of admission, with refractory and severe lactic acidosis.

### 3.2 Review of MTDS type 3 cases from the MENA region

We reviewed the clinical presentation, outcome, and genetic diagnosis for all 48 MENA region patients with MTDS type 3 who have been described (Table 2). In terms of ethnic origins, the reported families were Israeli Druze (Mandel et al., 2001), Algerian (Labarthe et al., 2005), Moroccan (Labarthe et al., 2005; Brahimi et al., 2009), Tunisian (Brahimi et al., 2009), Saudi (Al-Hussain et al., 2014), Egyptian (AlMenabawy et al., 2022), and Lebanese (reported in 2005 by Mancuso et al. and in the current report). Patients with this rare disorder have been reported from only 7 of the 21 countries in the MENA region.

**TABLE 2:** Clinical, biochemical, and molecular profiles of mitochondrial depletion syndrome type 3 patients from the MENA region. Abbreviations: N, number of patients; CG, consanguinity; ^, lost to follow-up; FTT, failure to thrive; HMG, hepatomegaly; NR, not reported; m, months; d, days; F, family; K, kindred; SNV, single nucleotide variant; P, pathogenic; LP, likely pathogenic; VUS, variant of unknown significance *familial variant suspected.

Population *Citation, year*	*Family*	*N*	*CG*	*Age of onset/death*	*FTT*	*HMG*	*Hepatic cytolysis*	*Cholestasis*	*Hypotonia/nystagmus*	*Lactic acidosis*	*Variant*	*Description/effect*
Israeli Druze Mandel et al., 2001	K1 F1–8	P1-15	+	<6 m/<1y	+	NR	+	NR	+/+	+	c.255del (p.Ala86fs)	nonsense/P
	K1 F2	P1-9	+	<6 m/<1y	+	NR	+	NR	+/+	+	c.255del (p.Ala86fs)	nonsense/P
	K2	P1-3	+	<6 m/<1y	+	NR	+	NR	+/+	+	c.255del (p.Ala86fs)	nonsense/P
	K3	P1	+	<6 m/<1y	+	NR	+	NR	+/+	+	c.255del (p.Ala86fs)	nonsense/P
Algerian Labarthe et al., 2005	F1	P1	-	1d/1 m	+	+	NR	NR	−/+	NR	c763_c766dup (p.Phe256Ter)	nonsense/P
		P2	-	4d/3 m	+	+	+	+	+/+	NR	c763_c766dup (p.Phe256Ter)	nonsense/P
Moroccan Labarthe et al., 2005; Brahimi et al., 2009	F1	P1	+	2d/3 m	+	+	+	+	+/+	+	c763_c766dup (p.Phe256Ter)	nonsense/P
		P2	+	15d/7 m	+	+	+	+	+/−	+	c763_c766dup (p.Phe256Ter)	nonsense/P
		P3	+	15d/22 m	+	+	+	+	−/+	+	c763_c766dup (p.Phe256Ter)	nonsense/P
	F2	P1	+	3 m/14 m	+	+	+	+	+/−	+	c.444–62C>A	SNV/VUS
		P2	+	3 m/3 m	+	+	+	+	+/−	NR	c.444–62C>A	SNV/VUS
	F3	P1	+	1d/2 m	+	+	+	+	+/+	+	c763_c766dup (p.Phe256Ter)	nonsense/P
Tunisian Brahimi et al., 2009	F1	P1	+	3 m/18 m	+	+	+	+	+/−	+	c.444–62C>A	SNV/VUS
Saudi Al-Hussain et al., 2014	F1	P1	+	8 m/11 m	+	+	+	+	+/−	+	c.223T>A (p. Trp75Arg)	missense/P
	F2	P2	+	1 m/8 m	+	+	+	+	+/+	+	c763_c766dup (p.Phe256Ter)	nonsense/P
	F3	P3	+	3 m/9 m	+	+	+	+	+/+	+	c763_c766dup (p.Phe256Ter)	nonsense/P
	F4	P4	+	1 m/1y^**^**^	+	-	+	+	+/−	+	c. 617G>A (p.Arg206Lys)	missense/P
Egyptian AlMenabawy et al., 2022	F1	P1	+	2 m/NR	+	+	+/+	+	+/−	-	c.255del/c.255del (p.Ala86fs)	nonsense/P
	F2	P1	_	6 m/NR	+	+	+/+	+	+/−	-	c.763_766 dup (p.Phe256Ter)	nonsense/P
											c.819 T > G p. (Phe273Leu)	missense/LP
Lebanese Mancuso et al., 2005; *this study*	F1	P1	+	1d/5 m	+	+	+	+	+/+	+	c.235C>T p. (Gln79*)	nonsense/P
		P2	+	2 m/4 m	+	+	+	+	+/+	+	c.235C>T p. (Gln79*)	nonsense/P
	F2	P1*	+	2w/1y	+	+	+	-	+/−	+	c.235C>T p. (Gln79*)	nonsense/P*
		P2	+	2 m/3 m	+	+	+	+	+/+	+	c.235C>T p. (Gln79*)	nonsense/P
		P3	+	1w/2 m	+	+	+	+	+/−	+	c.235C>T p. (Gln79*)	nonsense/P
Total (frequency)		48	45/48 (94%)	<6 m/<1y (98%)	48/48 (100%)	43/44 (98%)	47/47 (100%)	42/43 (98%)	46/48 (96%) 38/48 (79%)	43/45 (95%)	7 variants	nonsense: 43/48 (89%) missense: 2/48 (4%)

The clinical presentation of all patients was characterized by severe failure to thrive and cerebro-hepatic involvement. Hypotonia was the most frequent neurological symptom (98%), whereas nystagmus was a less constant finding (79%). The majority were diagnosed before 6 months of age and died before 1 year of age. All five reported Lebanese patients exhibited early-onset hepatic and neurological symptoms, before 2 months of age. Intrauterine growth retardation, low birth weight, and subsequent failure to thrive were present in all three patients. Outcomes were poor and death occurred within 5 months of age, with the exception of one patient who died at 11 months of age.

The biochemical hallmark was lactic acidosis associated with hepatic cytolysis, hypoglycemia, and cholestasis in all patients. Serum alpha-fetoprotein levels were elevated, in parallel to hypertyrosinemia, in all patients in the Israeli Druze cohort, two Saudi patients (F3, F4), and the three Lebanese patients reported in this study.

Genetic profiling of MTDS type 3 patients reported in the MENA region revealed a total of seven variants, mostly nonsense mutations (89%) (Table 2). The most frequent variant was c.255del (p.Ala86fs), in the Israeli Druze cohort, followed by another founder mutation c763_c766dup (p.Phe256Ter) in the Moroccan and Tunisian patients; this also occurred in Egyptian and Saudi patients. All Lebanese patients (those described by Mancuso et al., in 2005 and in the current report) carried a unique nonsense mutation c.235C>T p. (Gln79*).

## 4 Discussion

The exact incidence or prevalence of MTDP type 3 in different populations is not yet known; however, this disorder seems to be more prevalent in the MENA region, and specifically in countries with high rates of consanguineous marriage, as in Lebanon (Barbourand Salameh, 2009).

The clinical presentation of the Lebanese patients fell under the severe early-onset type of *DGUOK* deficiency, with rapid hepatic and neurological deterioration, as reported in other patients carrying null mutations (Dimmock et al., 2008). Clinical signs of prenatal mtDNA depletion, including microcephaly, low birth weight, and intrauterine growth retardation, all predictors of poor outcome and early death, were present in the Lebanese patients (Jankowska et al., 2021).

Hereditary tyrosinemia type 1 was suspected in view of the elevated plasma tyrosine levels in all three patients; however, the association of lactic acidosis with signs of liver failure and coagulopathy was mostly suggestive of mitochondrial disorders. The final diagnosis could not be confirmed in Patient 1 due to the unavailability of genetic testing at time of presentation. Ten years later, the two other affected family members were diagnosed, and the diagnosis confirmed by whole-exome sequencing, 1 month after their initial presentation. Subsequently, a retrospective diagnosis of MTDS type 3 was applied for Patient 1. This highlights the importance of whole-exome sequencing as diagnostic tool even in developing countries, such as Lebanon, shortcutting the diagnostic odyssey and the need for invasive and more expensive testing (Salman et al., 2022).

The prognosis for this devastating disease remains circumspect, since liver transplantation is the only therapeutic option. Determination of the timing and selection of patients for liver transplant remains difficult, especially in the case of the severe phenotype associated with the Lebanese mutation and its dismal prognosis. Liver transplantation would be indicated in patients without or with minimal neurologic involvement (Jankowska et al., 2021), although the evidence in the literature is limited in such cases. Regardless of the result of genetic testing, the decision for liver transplant is sometimes made depending on the clinical condition of the affected patient. None of our patients could be offered a liver transplant, in view of their early onset (Shimura et al., 2020) and severe neurological presentation (Squires et al., 2014).

With advances in next-generation sequencing and increased awareness among physicians, early identification and diagnosis of rare disorders such as MTDS type 3 has become possible. To date, known pathogenic variants in the *DGUOK* gene, associated with MTDS type 3, account for 36 out of 57 mutations. While patients carrying pathogenic missense mutations in *DGUOK* gene do not exhibit a clear genotype–phenotype correlation (El-Hattab and Scaglia, 2009), those with null mutations show a severe phenotype with poor outcome (Dimmock et al., 2008).

The nonsense mutation c.235C>T p. (Gln79*) in the *DGUOK* gene, considered to be a pathogenic variant according to ACMG and ClinVar, was found in all Lebanese patients upon next-generation sequencing. According to the sequencing criteria employed at Centogene, this variant was of good quality; thus, confirmation by Sanger sequencing was not warranted. A recent report by Arteche-López et al. (2021) confirms that Sanger sequencing is not needed as an internal control in the case of a high-quality single-nucleotide variant. The c.235C>T p. (Gln79*) mutation in the *DGUOK* gene creates a premature stop codon, resulting in a loss of function of *DGUOK* enzyme activity, thereby impairing the mitochondrial DNA salvage pathway with subsequent mtDNA depletion.

This *DGUOK* gene variant, unique to Lebanese patients and as yet unreported in any other population, may be considered a founder mutation in Lebanon. Further delineation of genotypic variants in populations with high consanguinity rates, as in the MENA region, is important for genetic counseling and pre-implantation diagnosis.

## Data Availability

The original contributions presented in the study are included in the article/supplementary material, further inquiries can be directed to the corresponding author.
